# The Perceived Ease of Use and Perceived Usefulness of a Web-Based Interprofessional Communication and Collaboration Platform in the Hospital Setting: Interview Study With Health Care Providers

**DOI:** 10.2196/39051

**Published:** 2023-01-23

**Authors:** Jason Xin Nie, Christine Heidebrecht, Andrea Zettler, Jacklyn Pearce, Rafael Cunha, Sherman Quan, Elizabeth Mansfield, Terence Tang

**Affiliations:** 1 Institute for Better Health Trillium Health Partners Mississauga, ON Canada; 2 Bellwoods Centres for Community Living Inc Toronto, ON Canada; 3 Accenture Toronto, ON Canada; 4 Department of Medicine University of Toronto Toronto, ON Canada

**Keywords:** health information technology, communication and collaboration, teamwork, design, technology acceptance model, qualitative method, communication, collaboration, hospital, care, team, professional, support, health information, technology, clinician, members, complex, lesson, education

## Abstract

**Background:**

Hospitalized patients with complex care needs require an interprofessional team of health professionals working together to support their care in hospitals and during discharge planning. However, interprofessional communication and collaboration in inpatient settings are often fragmented and inefficient, leading to poor patient outcomes and provider frustration. Health information technology can potentially help improve team communication and collaboration; however, to date, evidence of its effectiveness is lacking. There are also concerns that current implementations might further fragment communication and increase the clinician burden without proven benefits.

**Objective:**

In this study, we aimed to generate transferrable lessons for future designers of health information technology tools that facilitate team communication and collaboration.

**Methods:**

A secondary analysis of the qualitative component of the mixed methods evaluation was performed. The electronic communication and collaboration platform was implemented in 2 general internal medicine wards in a large community teaching hospital in Mississauga, Ontario, Canada. Fifteen inpatient clinicians in those wards, including nurses, physicians, and allied health care providers, were recruited to participate in semistructured interviews about their experience with a co-designed electronic communication and collaboration tool. Data were analyzed using the Technology Acceptance Model, and themes related to the constructs of perceived ease of use (PEOU) and perceived usefulness (PU) were identified.

**Results:**

A secondary analysis guided by the Technology Acceptance Model highlighted important points. Intuitive design precluded training as a barrier to use, but lack of training may hinder participants’ PEOU if features designed for efficiency are not discovered by users. Organized information was found to be useful for creating a comprehensive clinical picture of each patient and facilitating improved handovers. However, information needs to be both comprehensive and succinct, and information overload may negatively impact PEOU. The mixed paper and electronic practice environment also negatively impacted PEOU owing to unavoidable double documentation and the need for printing. Participants perceived the tool to be useful as it improved efficiency in information retrieval and documentation, improved the handover process, afforded another mode of communication when face-to-face communication was impractical, and improved shared awareness. The PU of this tool depends on its optimal use by all team members.

**Conclusions:**

Electronic tools can support communication and collaboration among interprofessional teams caring for patients with complex needs. There are transferable lessons learned that can improve the PU and PEOU of future systems.

## Introduction

### Background

Patients with complex care needs admitted to hospitals often require the services of an interprofessional team of health professionals working together to support their care [1]. However, in inpatient settings, interprofessional communication is often fragmented and inefficient [2,3]. Poor communication and teamwork can contribute to poor patient outcomes, such as delayed discharge, medication errors, and adverse and sentinel events, including death [4-7]. It can also lead to frustration among health care providers [8], especially when the providers are not on the same page regarding the plan of care [9,10].

Health information technology has the potential to improve interprofessional communication in hospital settings. Communication technology tools that are used vary between and within institutions and can range from numeric pagers to mobile devices or specialized software applications with varying degrees of integration with electronic health records [11-15]. Common concerns with existing technology include lack of context and structure, interruptive nature, privacy and security concerns, and lack of visibility to the entire care team [12,15-20]. The information required to best address a patient with complex care needs may also exist in a combination of paper and disparate electronic systems, resulting in various team members being unaware of or unable to access information critical to providing the best quality of care in a timely and efficient manner. Systematic reviews published in 2012 and 2019 highlight the lack of high-quality evidence on the effectiveness of current communication tools in hospital setting [12,14]. Moreover, there are concerns that these technologies are not optimally designed, and their use may further fragment communication and increase the demand on clinicians without demonstrating benefits [21].

To generate transferrable lessons that may improve the design of future health information technology solutions aimed at facilitating communication and collaboration between clinicians of interprofessional teams within hospitals, we performed a secondary analysis [22,23] of qualitative data collected as part of a mixed methods evaluation of a co-designed interprofessional communication and collaboration tool [24]. Results from the mixed methods study showed improved teamwork (encompassing both communication and relational aspects) in one of the two study wards after the introduction of the tool, without meaningful changes in face-to-face communication patterns during team rounds or adverse events in both wards. There is potential for an electronic tool to improve teamwork and communication, but success is dependent on the complex interactions of technological and nontechnological factors [24]. The focus of this paper is to analyze our qualitative data using Technology Acceptance Model (TAM) to understand clinicians’ perspectives on the tool’s perceived usefulness (PU) and perceived ease of use (PEOU) to generate lessons relevant for the design of future interventions.

### An Overview of the Electronic Communication and Collaboration Platform

To improve communication and collaboration among the interprofessional teams at the hospital, our team used agile methodology and co-designed a web-based technology platform with frontline clinicians using a variety of design methods as described by Tang et al [24,25]. It addresses issues with handoffs (with a physician sign-out tool), interprofessional collaboration (through the interprofessional care planner where information relevant to the team from each discipline can be viewed in one place and the patient flow planner in which barriers to discharge are identified and tracked), and team communication (secured team messaging that is attached to a patient and viewable by the entire care team). It also evolved to include an electronic discharge summary and an associated patient-oriented discharge summary to facilitate care transitions. Although the focus was on communication and collaboration, a progress note module (where typed notes can be generated and printed for the paper chart) was also developed to facilitate workflow and reduce double documentation.

**Figure 1:**
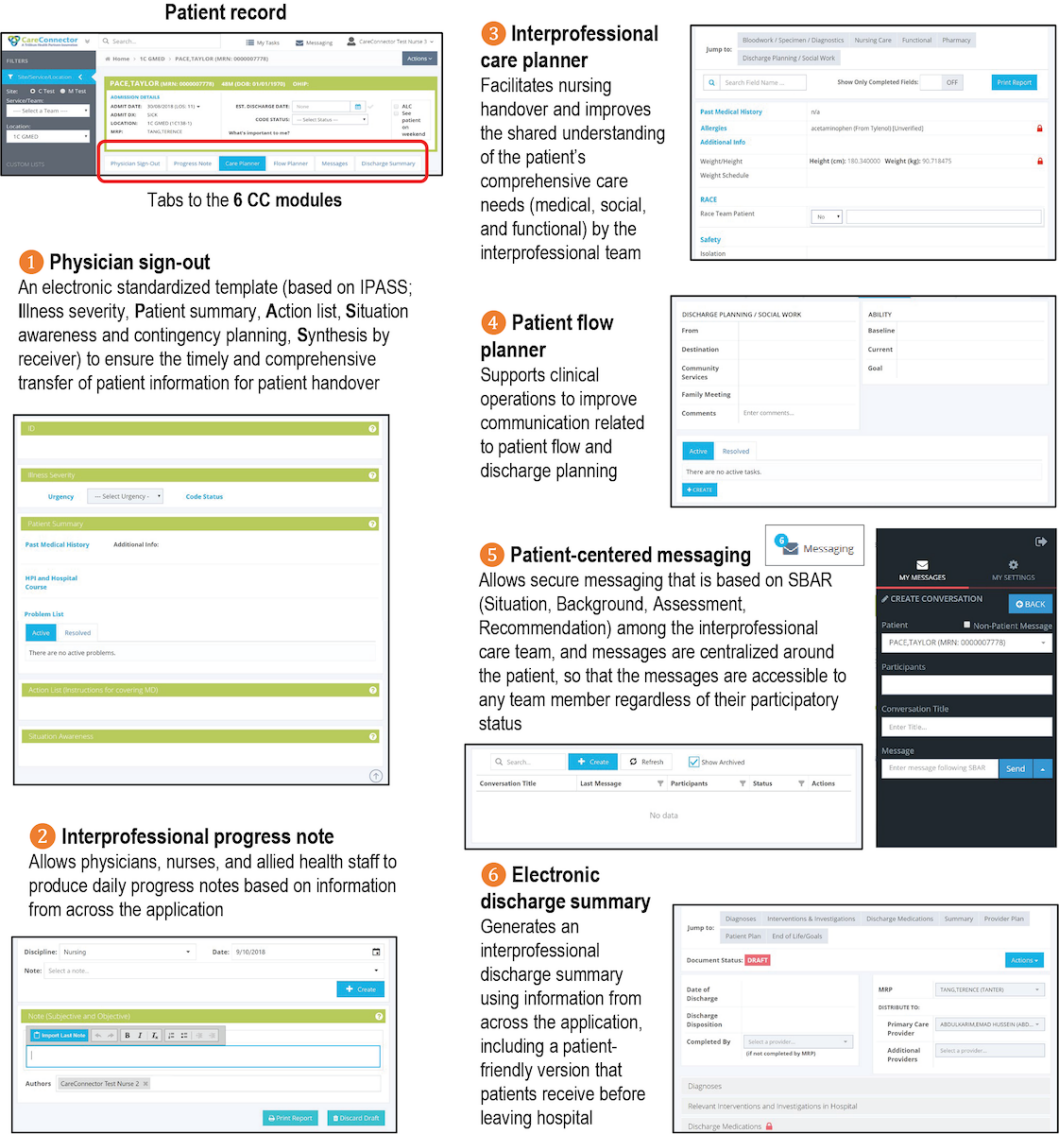
Care Connector modules and functionality. CC: Care Connector.

The tool is a web-based platform that, although distinct from the hospital’s primary vendor health information system (HIS), can retrieve information from and write information to the primary HIS using Health Level Seven, a technical standard that allows health-related information to be exchanged between health care applications [26]. It does not replace but augments the HIS by providing communication and collaboration features designed to fit the clinician workflow. The architecture of the Care Connector is modular, allowing each module to be developed independently while addressing different yet interconnected clinical workflows. The 6 key modules (Figure 1) are interconnected with each other and with the primary HIS by sharing information, thereby allowing for continuity of information (as changes in one module are reflected in other modules and in the HIS in real time), reducing the need for repeated data entries or the likelihood of missed information. It also allows information reuse (eg, past medical history captured in the physician sign-out is reused in the interprofessional care planner and discharge summary) to improve communication and reduce documentation effort.

### Technology Acceptance Model

The TAM, first developed in 1985 by Fred Davis, is used to provide a theoretical basis “of the effect of system characteristics on user acceptance of computer-based information systems” [27,28]. The TAM theorizes that actual system use is determined by a potential user’s attitude toward using the system, which in turn is based on the following 2 key beliefs: PU and PEOU [29]. PU is defined as “the degree to which an individual believes that using a particular system would enhance their job performance.” PEOU is defined as “the degree to which an individual believes that using a particular system would be free of physical and mental effort.” Moreover, PEOU is hypothesized to have a causal effect on PU because the easier a system is to use, the more useful the user will find the system [29].

## Methods

### Study Design

A secondary analysis was performed on the qualitative component of a mixed methods study conducted between February 2016 and July 2017 to assess the impact of an electronic communication and collaboration tool on communication, teamwork, and adverse events [24].

### Ethics Approval

Ethical approval was obtained from the Research Ethics Board of Trillium Health Partners (approval number: ID#691).

### Participants and Setting

Trillium Health Partners is one of the largest community-based hospital systems in Canada with 1306 beds across 3 sites. An electronic communication and collaboration tool, described in the section above, was implemented in 2 of the 5 General Internal Medicine wards at the Credit Valley Hospital site. Nurses and allied health care staff were ward based, whereas physicians who provided care to patients were dispersed throughout the hospital. At the time of the study, patient information was split between the hospital HIS and paper charts where progress notes and documentation were noted. In our mixed methods study, we recruited a diverse sample of frontline health care personnel using a purposeful maximum variation sampling strategy [30]. Potential participants in clinical and logistical roles in the 2 General Internal Medicine wards where the electronic tool had been deployed and used for at least 6 months were invited to participate.

### Data Collection and Analysis

CH recruited, acquired consent, and interviewed all the participants. A copy of the full interview guide is presented in Textbox 1. EM, TT, CH, AZ, and JXN were engaged in the analysis. An inductive approach was used in this study. Three researchers (AZ, TT, and CH) independently reviewed a purposive sample of 4 transcripts and, during a series of meetings, developed a coding framework. Subsequently, 2 (CH and AZ) researchers coded all the transcripts, with each member being the primary coder for half the transcripts, and second coded the other half to ensure that the codes were applied appropriately and consistently. The team resolved issues, came to consensus via discussions and meetings, and then reviewed the coded data and identified key themes. CH sent all the participants an email of a summary of major findings for member-checking to which no participant objected. All authors contributed to the writing of the manuscript.

**Interview guide.**

Background information

- Tell me a bit about your work and clinical role at the hospital.
- What other experiences have you had working with communication systems similar to Care Connector?
- How comfortable are you with information technology in general?
- How long have you used Care Connector?
- How often do you interact with Care Connector? (i.e. daily; per shift; weekly)
  - When in the day, or during your shift, do you tend to interact with Care Connector?
- When do you tend to interact with other care providers to make plans for patient care? Do these interactions involve Care Connector?

Impact of Care Connector on workflow, patient care, and interprofessional relations

- What modules do you primarily use?
- What gaps do you see Care Connector as addressing? (quality of patient care; interprofessional communications, patient handover, workflow efficiency etc.)
- Has Care Connector affected your workflow? If so, how? Provide an example/story illustrating this.
- Has using Care Connector affected patient care? If so, how? Provide an example/story illustrating this.
- Has Care Connector affected your workflow? If so, how? Provide an example/story illustrating this. Has using Care Connector affected patient care? If so, how? Provide an example/story illustrating this.
- Has using Care Connector affected your communications with other (physicians/nurses/allied health professionals/unit clerks/flow team members: insert appropriate role depending on interviewee’s role)? If so, how? Provide an example/story illustrating this.
- Has using Care Connector affected your communications with other team members? *[Specify physicians, nurses, allied health professionals unit clerks/flow team members, excluding the interviewee’s role, which has been covered above]* If so, how?

- Has Care Connector affected the relationship between staff/health care professionals? If so, how?
- Has Care Connector affected teamwork between you and your colleagues? If so, how?
- How do you feel about teamwork between you and other (choose discipline depending on role of respondent: physicians/allied health/nurses/etc.)? What about with other hospital staff?
- What are your thoughts on the effectiveness of care rounds?
  - “Does Care Connector support you in any way at care rounds?
  - Describe and get their feedback on the idea of the marketplace

Strengths and challenges of working with the new Care Connector modules

- How did you find the process used to introduce, implement and obtain feedback about Care Connector?
  What worked well?
  What could be improved?
- What features of the Care Connector modules do you find most useful?
- What features of the Care Connector modules need improvement?
- What challenges from a workflow and clinical documentation perspective has using Care Connector created, if any?
- If there were times when you had a choice between using Care Connector and completing a task using a conventional approach (e.g. when documenting progress notes), what made you choose Care Connector over the traditional approach or vice versa?
- Are there any unintended benefits or consequences you discovered from using Care Connector?
- Why or why not should Care Connector be introduced to other departments and hospital units?
- What are some other healthcare settings where Care Connector might be useful?
- If there were to be a module, or multiple modules, that would involve patients – and would facilitate communication between team members and patients themselves – do you think that that would be valuable?

Conclusion

- Is there anything else that you would like to comment about that I haven’t asked you about?

### Secondary Analysis

Our team performed a secondary analysis of all the original transcripts using the TAM lens by mapping questions from the original interview guide that were relevant to the TAM model (Textbox 1). Interview questions related to PEOU included the following: “What features of the technology need improvement?” “If there were times when you had a choice between using the platform and completing a task using a conventional approach, what made you choose the platform over the approach or vice versa?” “What challenges from a workflow and clinical documentation perspective has using the platform created, if any?” The participants were asked which features or functionalities were easy or difficult to use. Responses were analyzed to identify comments related to PEOU (eg, confusion, frustration, ease, difficulty, and intuitiveness). Regarding PU, we asked the following questions: “What modules do you primarily use?” “What features of the technology do you find most useful?” “What gaps do you see the platform addressing?” “Has the technology affected your workflow?” Finally, we specifically explored the perceived role of technology in facilitating teamwork and communication in team-based care as part of understanding PU. The questions included the following: “Has using the technology affected your communications with other team members?” “Has the technology affected the relationship between healthcare providers?” “Has the platform affected teamwork between you and your colleagues?” “Has using the platform affected patient care?”

A thematic content analysis approach was applied [31]. Our coding methods have been described by Tang et al [24]. Key themes and relationships between the themes were identified inductively through team members’ individual reviews and group dialogue regarding code reports and memos. Themes related to the PEOU and PU of the TAM were used for the analysis.

## Results

### Overview

**Figure 2:**
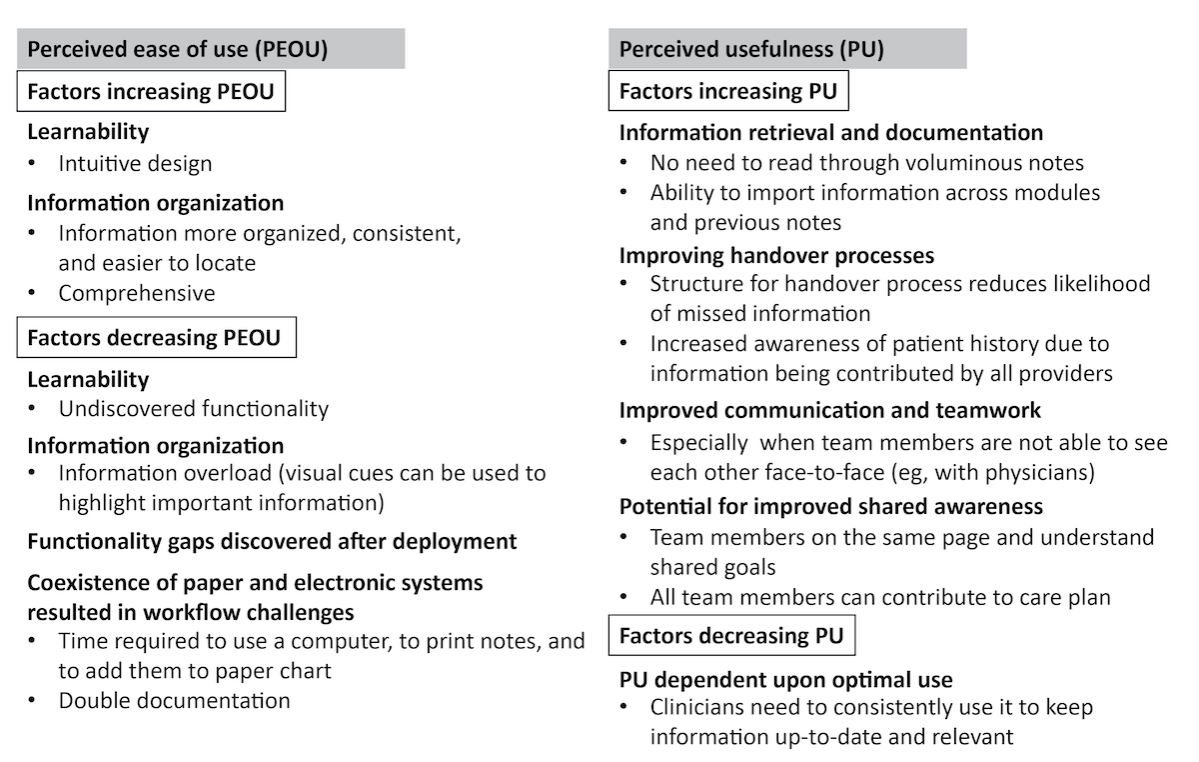
Hierarchy of themes.

In total, 15 transcripts were included in this secondary analysis, including the perspectives of physicians (4/15, 27%), nurses (5/15, 33%), allied health care professionals (4/15, 27%), and nonclinical support personnel (2/15, 13%). Here, we report the findings of our secondary analysis from the perspective of the TAM (Figure 2). Using this focused analytic approach, the following themes emerged in relation to PEOU: learnability, information organization, functionality gaps discovered after deployment, and challenges related to the coexistence of paper and electronic systems. The following themes emerged in relation to PU: efficiency in information retrieval, improved handover processes, improved communication and teamwork, and the potential for improved shared awareness.

### Perceived Ease of Use

With respect to PEOU, the following themes emerged: learnability, information organization (the need to achieve comprehensiveness without causing information overload), functionality gaps, and the challenge of the coexistence of paper and electronic systems.

#### Learnability: Trade-off Between Intuitive Design and Need for Learning

Through the agile software development methodology and continuous user engagement involved in its development [25], the platform was designed to be inherently intuitive, requiring minimal user training. Our findings indicated that these objectives were achieved; overall, the participants found the electronic communication and collaboration tool easy to use and adopt:

Yeah, I think it was pretty easy to pick up. You know it’s not hard to learn. And I think just a colleague of mine just showed me and it was fine.

Physician 1

The [implementation] of [the electronic tool], for me, I felt like it wasn’t too bad on learning stuff. It was pretty straightforward in terms of accessing, updating the information on there.

Nurse 4

However, the ease of use could be hindered if the users had not discovered a particular system functionality. For example, a patient’s medical condition in the past medical history section were captured discretely on the platform (ie, requiring each condition to be entered into a separate row) to facilitate information reuse throughout the platform. Users had the option of clicking the “Add” button to add a new row to the list, but the electronic tool also allowed the user to simply hit the *Enter* key to get to the new line so that users were not slowed down by using the mouse when they had to enter a long list of medical conditions. However, users who were unaware of this feature found the system to be labor intensive:

It takes forever to type all these things out and you have to do it for each patient. And, I think each time you have to click something on the screen to make something happen, it just increases the amount of work you have to do. So, for example, the past medical history section has this, like, Add thing where you do one past medical history, add each one at a time. I would never use that…Whatever it is, I’m not going to keep going back and pressing “Add”. It seems to be a cognitive load I don’t need to deal with.

Physician 4

#### Information Organization: Tension Between Comprehensiveness and Information Overload

The participants had different perceptions regarding the PEOU of the layout and information organization of the platform. The participants felt that the information in the electronic tool was more organized and consistent. Moreover, participants found that information that is well organized and easy to locate is useful for creating a comprehensive clinical picture of each patient and facilitating improved handovers, demonstrating that improved PEOU is associated with increased PU:

Things are less easily missed perhaps...[In the electronic tool] you always have a consistent layout, and people tend to put information in the same area. So you know hopefully that you’re not going to miss a piece of information elsewhere, because it’s more consistently used amongst nursing staff.

Nurse 3

I just feel that people are, it just seems to be more clear in the documentation being done in the [the electronic tool], I can’t explain why that is but it just is...The plan is better organized or the next steps are better listed.

Allied health 4

However, electronic systems can contribute to information overload, and important information and day-to-day changes can sometimes be overlooked. Some participants suggested that using headings better, reducing the amount of scrolling, and highlighting key information or changes may improve PEOU. For example, a physician observed that a key component of the daily progress note, the physician’s impression and plan for the day, is sometimes difficult to locate because of content that was copied forward:

When I see progress notes that have come out of [the electronic tool], the problem I always have is that they all look the same and they don’t highlight the day’s problems as well...it’s sort of hidden in the body. You have to sort out what’s changed and the problem list still stays the same very often either because people don’t want to change it or again, there just wasn’t enough to change. It always involves sort of hunting and trying to see what is different in today’s note versus the note that was written yesterday, and trying to find the data that looks different to find out what happened.

Physician 2

Similarly, another participant found that the tool could be improved by more prominent visual cues to highlight important information, especially for patients with complex medical needs:

I feel like the whole layout when you first open it and you have to like scroll through looking at all the different aspects, like where they’re from, if they’re diabetic, like how they take their meds [...], I feel like it’s so like...how do I describe this...like one colour, like nothing really stands out, I feel like it looks so...like, not blah, but it’s hard to find things if you’re trying to scroll through there fast. I feel like it could be more like friendly, like maybe more colours or like the way it’s laid out.

Nurse 2

#### Functionality Gaps Discovered After Deployment: Potential to Improve PEOU

Several participants identified functionality gaps and workflow requirements that were previously unaccounted for, which limited the PEOU of the electronic platform. For example, the messaging component was a core feature of the system that allowed users to send messages to any member of the care team by name. However, clinicians might not always know the name of the team members they were sending the message to, but they did know the provider role they were trying to connect with. One participant suggested that having the added functionality to send a message to select roles within the patient’s care team would improve PEOU:

Well, I mean, I’m not completely clear on who I can send a message to. But I’m assuming you can send it to the allied health team but, you know, there is no part on [the electronic tool] which identifies who the allied health person is that may be following the patient. So if that was identified then it would make it much easier to send a message to them.

Physician 4

In addition, the ability to search the system for patients by their names was also a suggested functionality that was not present in the original implemented system:

It’s just helpful if I just am able to just search the patient’s name and then get their information [the patient’s chart]. Because right now what I’m doing is I’m clicking on every team and just seeing whether or not they [the patient] were on those teams.

Nurse 1

#### Coexistence of Paper and Electronic Systems Resulted in Workflow Challenges

The clinical practice environment of our organization at the time when the study was conducted was a mix of paper and electronic systems that clinicians had to navigate. Clinical documentation (eg, notes) was paper based, while some information (eg, vital signs and diagnostic testing results) was captured electronically. To reduce double documentation (ie, having users enter the same information both on paper and electronically on the communication tool), a co-designed feature of our system allowed users to efficiently generate documentation electronically. However, owing to the practice environment, this electronically generated documentation still needed to be printed and placed in paper charts. This administrative burden caused frustration for users and significantly limited the PEOU. Some users weighed the cost and benefit of the extra effort required for printing and reported that they would only use the tool when the benefits outweigh the time and effort required:

I think if I didn’t have to print the notes out and then put it in the chart, that definitely [would make me more likely] to chart things on the computer. Yeah, I think the main thing is I have to get the chart anyways, so sometimes it’s much faster for me to just scribble notes in the chart, whereas with [the electronic tool] I have that extra step of finding a computer, print it, find a printer, print, and then find the chart and putting it in the chart.

Physician 1

In a mixed paper and electronic environment, participants often chose the method that was efficient for them in the moment for a particular task:

So one example is, for example, so if I have a longer note to type, like a family meeting that I need to document, I would probably use [the electronic tool], just because it’s a longer note and it would require more handwriting, if I were to write it out. So I would choose [the electronic tool] to document longer progress notes. In terms of handwriting, if I were to physically write in the chart it would be something very short.

Allied health 2

In addition, although the system was designed to reduce double documentation, the paper documentation requirement had made this unavoidable in some situations. For example, an allied health professional expressed frustration with a specific assessment form that was not supported by the electronic tool:

I think one of the things specifically to me that I find a little bit frustrating in my work is kind of the double charting that we do. So basically we have an [assessment form] that we fill out for every new assessment that has all the information on the patient’s background and then what we found in the assessment and what our recommendations are. And then in addition to that we also do a chart note. So when I get a new assessment I have to do a new chart note, I have to do [an assessment form], I have to do an order in the chart. And I have to put a sign above their bed with my recommendation. So it’s a lot of double charting or double writing.

Allied health 1

### Perceived Usefulness

Regarding the PU and the role of technology in supporting teamwork and communication, the following themes emerged: efficiency, improved handover, mode of communication (electronic tools play a role when face-to-face communication is not possible), degree of use (usefulness depending on extent of use), and shared awareness (even in the absence of direct communication).

#### Efficiency in Information Retrieval and Documentation

The care planning module (distinct from documentation) of the electronic tool made it easier to retrieve information for care and planning. This section of the tool was primarily used by nursing and allied health care staff members. Key information necessary for care planning and decision-making was well organized under clear section headings without the need for reading through voluminous documentation, thus saving time and increasing efficiency. Allied health care personnel, who often found following physician notes challenging, appreciated that information was organized around headings that were relevant to care planning, which helped to make the information easy to find and actionable:

And I think that (electronic tool) gives a standardized format of how to, I mean (certain allied health disciplines) tend to have a standardized way of documenting whereas I find the physicians not always. So I find that...to read what their plan is...is easier.

Allied health 4

It shortens the time that I have to spend digging for information because I have information readily accessible and available in some degree.

Nonclinical support 1

It’s just better…the information is definitely more organized. And just the key things that we’re looking for it’s just...they’re all included in [the electronic tool], so it’s just easy for us to communicate.

Nurse 1

Efficiency also increased through features supporting general documentation, including the ability to import information across different modules of the system and previous notes, as noted by a user:

I think that I have enjoyed the efficiency that it’s given me, and particularly with that import last note function, and I think a lot of us have used that because essentially we’re assessing the same sorts of things with patients every time we see them. We’re just updating, you know, their new functional status. And so it takes a lot of time to rewrite all of that, or if there’s specific things about the patient’s background or history that you want to mention, you don’t have to retype or rewrite all those things. So I think from an efficiency perspective, especially with the import last note function, it’s given me a lot more efficiency. For me, I can see a lot more patients because I’m not handwriting notes all day. So I can type faster than I can handwrite. So just from that perspective that’s been nice.

Allied health 1

#### Improving Handover Processes

Participants uniformly perceived the electronic tool to be especially useful during handover (which occurred when physicians rotated off clinical service or provided weekend coverage and when nurses changed shifts). They perceived the communication tool as providing a structure for the handover process and reducing the likelihood of missed information:

It provides a better hand over than we were doing before. I would always worry—I mean I tended to be pretty thorough in my emails and that but you would always worry that there were details that were missed, and email is just free form so it’s nice to have the organization the way it is now in terms of their past history, their issues and then the problem list, and so I think that’s probably a safer way to ensure that relevant information gets passed on.

Physician 2

Another participant highlighted increased awareness of patient history because information in the tool was contributed by all previous providers rather than just from the previous shift, making the collective knowledge of the patient available:

There’s a way for information to be passed on not just between nurses that are handing over but from prior nurses as well, because we can provide historical information on there to guide care. So I think there is more continuity in terms of information being passed forward, not just based on one shift’s information, but the information coming from many nurses prior to that.

Nurse 3

#### Improved Communication and Teamwork With Team Members Not Physically Present on Unit

At our institution, physicians attended to patients in many different wards, whereas nursing and allied health care teams were assigned to one ward. Participants observed that face-to-face communication when engaging in active care planning is preferred whenever possible. Therefore, the electronic tool was particularly useful for communication and facilitating teamwork when team members are unable to see each other face-to-face. Allied health care participants commented on the improved quality of communication with physicians as they may not always be on the ward:

I feel like [the electronic tool] might have made [teamwork] easier with the physicians.[...] We can also just look at what the physicians have written about the patient and their plans which can also limit the amount of time nursing is paging the physicians going, you know, “What do you want to do with this?” when they’ve probably already written it somewhere.

Nurse 2

[The electronic tool] sort of started to address the communication issue that we all sort of seem to have, communicating with the physicians I guess is what I’m referring to most. Again, because they’re not always on the unit, whereas the other staff, if we need to communicate with them, we can usually find them pretty easily.

Allied health 1

#### Potential for Improved Shared Awareness Among Interprofessional Team Members

Participants reported that distinct from the ability to facilitate direct communication (eg, via messaging), the designed system was useful for teamwork and collaboration because it improved shared awareness among the team. Participants noted that as all team members had access to the tool, it was easy for all team members to be “on the same page” and understand shared goals for the patient:

In a way, yes [the electronic tool addresses gaps in respondent’s work], because at least everyone that’s involved with the patient has access to it, so instead of it just being me trying to make those adjustments—you know, like allied health they have access and they can make those changes as well—so that definitely helps to put all the connections into place and stuff.

Nurse 4

Moreover, the Care Planner module allowed all team members to contribute to the patients’ care plan. Understanding and contributing to the shared goals for the patient was identified as having the potential for more efficient discharge planning:

It’s definitely improved communication. I know that some of the social workers, patient flow and, [nursing], we communicate through the [Care Planner]. So it just helps improve communication and then the discharges happen faster. There’s not a lot of gaps that we’ve missed with regards to discharge planning. So it definitely fills those communication gaps.

Nurse 1

Although participants have outlined the many positive benefits of an electronic communication and collaboration system, PU is dependent on optimal use by all clinicians. When clinicians consistently use it, information is up-to-date and relevant. However, if this is not the case, the PU of the system will decrease as echoed by a participant:

[My] only wish is [that] all the physicians were updating it. Because you know, some of them are better than others. Some of them are, you know, like updating daily or every other day or, you know, putting some extra notes and taking summaries...but if only all of them were updating on a regular basis then that would be helpful.

Nonclinical support 1

## Discussion

### Principal Findings

This study explored clinicians’ perspectives on the PEOU and PU of a web-based electronic communication and collaboration platform designed to facilitate team-based care for hospitalized patients with complex needs. Our results demonstrate a number of transferable lessons for others designing and implementing health information technology aimed at facilitating team communication and collaboration for inpatient care.

The design goal of the platform was to be intuitive to users requiring minimal documentation or training. However, there are often trade-offs in the design of an intuitive user interface [32]. The situation experienced by our user, who was frustrated with having to click the “Add” button to add a text field while failing to recognize that pressing *Enter* key would do the same thing, highlighted trade-offs between affordance (the intuitiveness of a visual element), learnability, efficiency, and discoverability. The *Add* button had high affordance, leading to higher platform learnability, but it decreased efficiency (ie, the need for mouse click). Pressing *Enter* key was a more efficient way of accomplishing the task, but it had low affordance (no visual element) and required users to discover this feature [32,33].

More information is not always better especially as it pertains to health information technology. Information overload in electronic health records has been shown to contribute to clinician stress and burnout, worsened workloads, and create opportunities for errors [34,35]. Although the users of our electronic platform reported that succinct information that was well organized and comprehensive made the system easy to use, the sheer amount of information captured on the platform could detract from the ease of use of the system [35]. This might be due to repetitive information in progress notes made possible by import features, causing “note bloat” (unnecessarily long cut-and-pasted progress notes) [35] and remains a tension to be considered in future system designs.

Functionality and workflow gaps that were not identified in the co-design of the platform emerged after clinical use. In our example, it was the inability to send messages to professional roles when the name of an individual team member was not known, and the inability to search patients by name. Therefore, it is critical to periodically evaluate functionality and workflow after implementation to identify areas of improvement that were not initially foreseen.

Clinical environments are complex, and the combination of paper and disparate electronic systems presents a unique challenge to system designers. Our study highlighted that in these blended environments, printing and double documentation are major issues that designers should seek to eliminate.

Our findings indicate that an electronic communication and collaboration system can achieve high usefulness with respect to improving efficiency and supporting improved handover when it is appropriately adopted. Participants in our study reported an improved quality of patient handover with the electronic platform, as information transfer was standardized and important details were not missed, ensuring continuity of patient care. This is consistent with previous literature in which electronic sign-out or handoff tools have been shown to improve the process, with fewer information omissions and improved efficiency [36-38].

Our results showed that clinicians see value in face-to-face interactions in care planning, and electronic tools may play a role in situations where these interactions are not possible. A previous study observed that non-IT communication was positively correlated with software adoption, suggesting that the relationship was not substitutive but rather complementary [39]. Our findings also suggest that the use of electronic platforms complements, rather than substitutes, in-person communication; it has strengthened electronic information exchange, especially across disciplines and during handover; however, face-to-face interactions remain highly valuable in active care planning.

Although the TAM suggests that PEOU and PU predict actual system use, our results also suggest that actual system use may impact PU. It is not difficult to see that for a teamwork and collaboration tool, the lack of users can be a big reason why it can fail to live up to its intended purpose. Our data reinforce that a shared communication and collaboration tool is most effective when all team members use it and keep information up-to-date, which leads to increased system use by other members of the team, and increased system use may further increase information quality. Conversely, the lack of system use by other team members adversely affects the PU for those who use it. The design of team collaboration tools should, therefore, also include looking at the groups (rather than individuals) who will be using the tool (eg, using collaboration personas) [40,41]. This also has implications for the implementation strategy, as a limited deployment of such tool may limit its usefulness.

Finally, our data suggests that electronic tools can play a role in supporting shared awareness among teams. Common ground—shared knowledge and understanding among ≥2 agents—plays an essential role in communication and collaboration within health care teams [42-44]. Discharge, care planning, workflow planning, rounds, and patient goal setting have been observed as care areas in which the establishment of common ground is critical [44]. In addition to trust in colleagues’ abilities—and knowledge of limitations in ability—Kuziemsky et al [44] identify the “push” and “pull” of information exchange as key elements in the establishment of common ground. Technology can be designed to support grounding through electronic channels when face-to-face interactions are not feasible [44]; we observed that many of the platform’s modules enhance information transfer to others (“pushing”). Respondents in our study described an increased ability to share detailed patient information with colleagues and provide input to others’ care plans. However, when tools are unidirectional, it is not always possible for users to obtain sufficient information about a patient or plan or to confirm that information has been received [44,45]. The patient-centered messaging module was bidirectional and thus had the potential to enhance information pushing when confirmation of receipt was required. It is also possible that once it is more widely used across disciplines, this module will increase the occurrence of electronic nonurgent information seeking (“pulling”).

### Strengths and Limitations

One of the strengths of our study is its focus on the communication and collaboration experience of interprofessional teams (as opposed to documentation or other usual health record functions) that are critical to the care of patients with complex needs. Our team members were involved in both primary and secondary analyses, thereby improving rigor [46]. This study was also carried out in a real-world busy suburban community teaching hospital, and findings will thus likely be relevant in a wide range of teaching and nonteaching environments. Few studies have examined the PEOU and PU of electronic collaboration and teamwork tools using a qualitative methodology [47,48], with a focus on transferrable lessons. However, our study does have several limitations. First, the tool studied is a home-grown tool designed at our institution, and many of our findings may not be generalizable to other settings. We mitigated this limitation by conducting a secondary analysis informed by TAM that allowed us to explore lessons of technology acceptance that are likely generalizable to other settings. Second, our study was conducted at a single institution in the hospital environment. The value of improved communication and collaboration may be greater in the community or across care settings and organizations. Recognizing this limitation, our team is actively working on applying the lessons learned from the hospital environment across care settings. Third, despite purposive sampling, most participants self-reported that they were very comfortable with the technology. Finally, this paper focuses on the TAM assessing PEOU and PU and does not take into account the complex organizational, cultural, and environmental contexts, which undoubtedly affect the use of technology in the health care setting. Despite its simplicity, the TAM is one of the most widely used frameworks in predicting information technology adoption, and it has shown validity and reliability in effectively assessing technology acceptance [49,50]. However, the simplicity of this model also receives substantial criticisms in that it oversimplifies the complexity of the sociotechnical system [51] by focusing only on individual users’ perceptions, beliefs, and intentions. Alternate approaches that recognize the complexity of issues surrounding the implementation of health information technology are required [51]. We chose this approach to clearly present lessons that may be important in the design of future tools. Looking at the factors of TAM using a qualitative approach also surfaces many of the organizational and social components (eg, mix of paper and electronic charts, provider workflow, and interruptions). We point readers to our published mixed methods paper that contains the nontechnology factors that we encountered in this project [24].

### Conclusions

Well-designed electronic tools that support the communication and collaboration needs of interprofessional teams are uncommon. To increase PEOU, future system designers should adhere to known usability principles relating to visual designs, consider the optimal training needs of users, ensure that information is succinct and organized, consider additional features that improve workflow, and remove logistical barriers if the system is embedded in a mix of paper and electronic systems. Users are likely to perceive the usefulness of these systems in their ability to increase efficiency, to support improved handovers, to allow communication and collaboration when face-to-face interactions are inefficient or impractical, and to promote shared understanding among team members. Owing to the collaborative nature of these tools, their actual use by all team members may impact the PU.

## Abbreviations

HIS: health information system
PEOU: perceived ease of use
PU: perceived usefulness
TAM: Technology Acceptance Model
